# Detection of Cytokines and Collectins in Bronchoalveolar Fluid Samples of Patients Infected with *Histoplasma capsulatum* and *Pneumocystis jirovecii*

**DOI:** 10.3390/jof7110938

**Published:** 2021-11-04

**Authors:** Laura E. Carreto-Binaghi, Eda P. Tenorio, Fernando R. Morales-Villarreal, El Moukhtar Aliouat, Edgar Zenteno, José-Arturo Martínez-Orozco, Maria-Lucia Taylor

**Affiliations:** 1 Laboratorio de Inmunología de Hongos, Unidad de Micología, Departamento de Microbiología-Parasitología, Facultad de Medicina, Universidad Nacional Autónoma de México (UNAM), Mexico City 04510, Mexico; lecarreto@iner.gob.mx; 2 Laboratorio de Inmunobiología de la Tuberculosis, Instituto Nacional de Enfermedades Respiratorias Ismael Cosío Villegas (INER), Mexico City 14080, Mexico; 3 Departamento de Bioquímica, Facultad de Medicina, UNAM, Mexico City 04510, Mexico; ep.tenorio@unam.mx (E.P.T.); ezenteno@unam.mx (E.Z.); 4 Departamento de Microbiología Clínica, INER, Mexico City 14080, Mexico; fernando62fmv@gmail.com (F.R.M.-V.); jarturoinfectologia@iner.gob.mx (J.-A.M.-O.); 5 CNRS, Inserm, CHU Lille, U1019-UMR 8204-CIIL-Center for Infection and Immunity of Lille, Institut Pasteur de Lille, University Lille, F-59000 Lille, France; elmoukhtar.aliouat-3@univ-lille.fr

**Keywords:** *H. capsulatum*, *P. jirovecii*, bronchoalveolar lavage, SP-A and SP-D, cytokines

## Abstract

Histoplasmosis and pneumocystosis co-infections have been reported mainly in immunocompromised humans and in wild animals. The immunological response to each fungal infection has been described primarily using animal models; however, the host response to concomitant infection is unknown. The present work aimed to evaluate the pulmonary immunological response of patients with pneumonia caused either by *Histoplasma capsulatum*, *Pneumocystis jirovecii*, or their co-infection. We analyzed the pulmonary collectin and cytokine patterns of 131 bronchoalveolar lavage samples, which included HIV and non-HIV patients infected with *H. capsulatum*, *P. jirovecii*, or both fungi, as well as healthy volunteers and HIV patients without the studied fungal infections. Our results showed an increased production of the surfactant protein-A (SP-A) in non-HIV patients with *H. capsulatum* infection, contrasting with HIV patients (*p* < 0.05). Significant differences in median values of SP-A, IL-1β, TNF-α, IFN-γ, IL-18, IL-17A, IL-33, IL-13, and CXCL8 were found among all the groups studied, suggesting that these cytokines play a role in the local inflammatory processes of histoplasmosis and pneumocystosis. Interestingly, non-HIV patients with co-infection and pneumocystosis alone showed lower levels of SP-A, IL-1β, TNF-α, IFN-γ, IL-18, IL-17A, and IL-23 than histoplasmosis patients, suggesting an immunomodulatory ability of *P. jirovecii* over *H. capsulatum* response.

## 1. Introduction

Reports on *Histoplasma capsulatum* and *Pneumocystis* sp. co-infection in wild animals [1,2] and humans [3,4,5,6,7,8,9] are scarce, especially in immunocompromised patients, and both mycoses are AIDS-defining conditions [10]. Pneumonia is the primary clinical feature of histoplasmosis [11] and pneumocystosis [12], although disseminated and chronic diseases frequently occur in histoplasmosis [13], but rarely in pneumocystosis [14,15].

*H. capsulatum* is a dimorphic fungus that causes histoplasmosis, one of the most common human respiratory mycoses. The aerosolized infective mycelial phase propagules of *H. capsulatum* are the source of infection for mammalian hosts in the environment and, in the infected host, the fungus converts into a parasitic and virulent yeast phase [16].

*Pneumocystis* species are opportunistic fungi that cause disease in several mammals and each fungal species infects only a specific host, a characteristic known as stenoxenism [17]; in humans, *Pneumocystis jirovecii* is the associated species [18]. All *Pneumocystis* spp. have trophic and asci forms, and the latter act as aerosolized propagules for transmission between individuals [19]. *H. capsulatum* is an intracellular facultative parasite, mainly within macrophages, while *P. jirovecii* is adhered preferentially on the surface of type 1 pneumocytes [20].

In the alveolar microenvironment, both pathogens interact with the surfactant proteins A and D (SP-A and SP-D), which are pulmonary collectins involved in microbial recognition and innate immune response [21,22,23]. McCormack et al. [24] described that collectins have fungicidal activity against *H. capsulatum* yeasts through protein biosynthesis inhibition, which leads to decreased yeast reproduction in the host cells. However, the exact mechanism for SP-A and SP-D binding to *H. capsulatum* is unknown. In contrast, SP-A and SP-D bind to *Pneumocystis* through the major surface glycoprotein and the cell wall β-glucan; however, none of these collectins induces phagocytosis of *Pneumocystis* [25,26]. During *Pneumocystis* infection, increased levels of SP-A and its mRNA were reported in alveolar cells [27,28].

Innate immunity mediates cellular effectors to sense the *H. capsulatum* mycelial phase infection and its subsequent thermo-dimorphic transition. After 2–3 h, the mycelial phase initiates its transition to the yeast phase in the upper and lower respiratory tract [29]. The innate immunity is necessary to orchestrate the initial inflammatory response. Type I interferon (IFN-I) is produced by bone marrow-derived macrophages after infection with conidia from the *H. capsulatum* mycelial phase [30]. IFN-I is also produced by a subset of lung conventional dendritic cells (CD103^+^) with the participation of the endogenous Toll-like receptors (TLR-7 and TLR-9), which are critical for the IFN-I response and the host survival to experimental histoplasmosis infection using the yeast phase [31]. Production of the chemokines CCL3 (MIP-1α), CCL4 (MIP-1β), CCL5 (RANTES), and CCL11 (eotaxin) is associated with innate and adaptive responses in mice inoculated with the yeast phase of the fungus [32]. In general, inflammatory molecules have been preferentially evaluated in experimental murine histoplasmosis using as inoculum the yeast phase, which is the fungal morphotype that remains along the adaptive immune response; however, this fact could mask the initial necessary steps for the innate response after a natural infection, where the host cells confront the mycelia to yeast transition. *H. capsulatum* infection induces the release of proinflammatory cytokines (mainly TNF-α, IL-1β, GM-CSF, IL-6, IL-23, and IL-17) [33,34] that activate macrophages and stimulate granuloma development through the production of IL-17 [35]. However, successful immunity against *H. capsulatum* infection leading to fungal clearance is controlled primarily by a Th1 adaptive response, whereas Th2 response contributes to a worse disease prognosis [36,37].

The immune response to *P. jirovecii* has been less explored and most of the data were obtained from mouse models using the *P. carinii* species (now renamed as *P. murina*), which suggests that the pathogen’s clearance correlates inversely with the type of inflammation [38,39,40]. The innate response mediated by IFN-I and TNF-α has a role in *Pneumocystis* pneumonia complications, which contributes to lung injury [41,42,43,44,45]. However, IL-1β and IL-6 recruit lymphocytes, macrophages, and neutrophils for *Pneumocystis* clearance [46,47]. *Pneumocystis* infection appears to be associated with dysfunction of the IL-23/IL-17 axis, with higher lung fungal burdens and reduced production of IL-17 and chemokines CXCL10 (IP-10), CCL3, CCL4, and CCL5 [48], all crucial for infection resolution [45]. Th1 cytokines also have a critical role in the clearance of *Pneumocystis* sp. infection [49,50]. IL-12 plays a vital role in *Pneumocystis* clearance, favoring inflammation through TNF-α and IFN-γ production by alveolar macrophages [51]. Regarding the Th2 response, reports have showed that IL-10-deficient mice develop more lung damage [52,53], highlighting the destructive role of excessive inflammation in *Pneumocystis* sp. infections.

The most important contribution and the aim of this pioneer study was the evaluation of the pulmonary immunological response in patients presenting acute pneumonia with *H. capsulatum*, *P. jirovecii*, or the co-infection with both fungal pathogens; thus, we analyzed the pulmonary collectin and cytokine patterns in their bronchoalveolar fluid. Considering these mycoses are described as AIDS-defining conditions and are frequently diagnosed in HIV/AIDS patients, this study included a population of HIV patients infected with both fungi.

## 2. Materials and Methods

### 2.1. Study Groups

We evaluated 131 individuals who participated in a previous report by Carreto-Binaghi et al. [9]; 104 of them were patients hospitalized for acute hypoxemic pneumonia at the Instituto Nacional de Enfermedades Respiratorias Ismael Cosío Villegas (INER) in Mexico City. In addition, eight healthy volunteers and 19 HIV patients without histoplasmosis or pneumocystosis were included as control groups (see Table 1). According to the medical staff, based on the clinical and radiological data, all patients required a bronchoscopic diagnostic procedure; samples were collected within the first 24 h of their hospital admission. Thus, no samples were obtained ex professo for this study. Patients were diagnosed with *H. capsulatum*, *P. jirovecii*, or the co-infection with both pathogens by sequencing their specific nested-PCR products obtained from the molecular markers Hcp100 protein [9] and the two mitochondrial ribosomal subunits [9], respectively.

### 2.2. Bronchoalveolar Lavage (BAL) Samples

BAL samples from patients admitted with pneumonia were collected at the Instituto Nacional de Enfermedades Respiratorias Ismael Cosío Villegas (INER), in Mexico City, CDMX, Mexico; BAL samples from healthy volunteers were donated by the Departamento de Investigación en Microbiología, INER. Details of the study groups are listed in Table 1. Within the first 24 h of admission, all individuals underwent a standardized flexible bronchoscopy procedure, performed according to the American Thoracic Society guidelines [54]. Briefly, the bronchoscope was placed in a wedge position within the middle lobe, 180 mL of normal saline solution were instilled through it and retrieved using a negative suction pressure (a sample was considered optimal if >30% of the instilled volume was retrieved). After collection, BAL samples were processed immediately at the Laboratorio de Inmunología de Hongos, Unidad de Micología, Facultad de Medicina, UNAM. Samples were centrifuged at 2800× *g* at 4 °C for 20 min, and each supernatant was aliquoted into 600 µL low-protein binding microcentrifuge tubes (Eppendorf North America, Inc., Hauppauge, NY, USA) and frozen in liquid nitrogen until collectin and cytokine determinations.

### 2.3. Compliance with Ethical Standards

This work was approved by the School of Medicine Research and Ethics Committee (UNAM, report 132/2015) and by the INER Ethics Research Committee (protocol B13-14). In accordance with the ethical standards of the Helsinki Declaration (1964, amended in 2013); each patient and volunteer signed a written informed consent before the BAL sample collection.

### 2.4. Collectin Determination

Human surfactant-associated proteins A and D (SP-A and SP-D) were quantified in duplicates using BioAssay ELISA kits (USBiological Life Sciences, Salem, MA, USA), following the manufacturer’s instructions. The ELISA assays detected a range of 5–100 ng/mL of SP-A and 6.25–400 ng/mL of SP-D. The absorbance values in both assays were measured with an Epoch microplate spectrophotometer (Biotek Instruments, Inc., Winooski, VT, USA) with a filter of 450 nm.

### 2.5. Cytokine Determination

Cytokines were quantified using the LEGENDPLEX Human Inflammation Panel (BioLegend, San Diego, CA, USA), following the manufacturer’s instructions. Panels containing a fluorescent dye, allophycocyanin (APC)-labeled beads conjugated to a monoclonal antibody specific for each target cytokine, were used to quantify IL-1β, IFN-γ, TNF-α, IL-5, IL-6, IL-10, IL-12p70, IL-13, IL-17A, IL-18, IL-23, and IL-33, as well as MCP-1 (CCL2) and IL-8 (CXCL8) chemokines. The panel detects cytokines with a high sensitivity of 0.6–2.1 pg/mL. Data acquisition was performed with a BD FACS Calibur dual-laser flow cytometer (BD Biosciences, San Diego, CA, USA) at the Laboratorio Nacional de Citometría de Flujo, Instituto de Investigaciones Biomédicas, UNAM. Flow cytometry data were analyzed with the LEGENDPLEX software v7.0 (BioLegend) to obtain the concentration of each cytokine in the samples. Assays were performed in duplicates.

### 2.6. Statistical Analyses

The Kruskal-Wallis test followed by Dunn’s multiple comparison test were performed using the GraphPad Prism software v9.00 for Windows (San Diego, CA, USA), and *p*-values ≤0.05 were considered significant. All groups of individuals were always compared to the control groups.

## 3. Results

**Figure 1:**
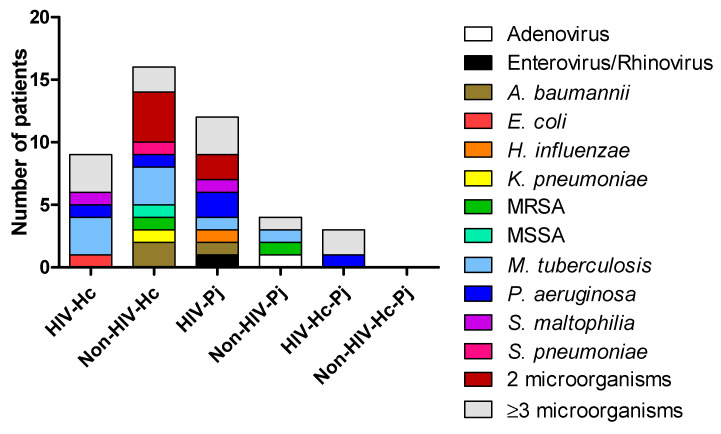
Diagnosis of concomitant infections in the studied patients. Other microorganisms were detected in the BAL samples by different methods: culture for bacteria and other fungi, and PCR for viruses. *A. baumannii*: *Acinetobacter baumannii*; *E. coli*: *Escherichia coli*; *H. influenzae*: *Haemophilus influenzae*; *K. pneumoniae*: *Klebsiella pneumoniae*; MRSA: Methicillin-resistant *Staphylococcus aureus*; MSSA: Methicillin-susceptible *Staphylococcus aureus*; *M. tuberculosis*: *Mycobacterium tuberculosis*; *P. aeruginosa*: *Pseudomonas aeruginosa*; *S. maltophilia*: *Stenotrophomonas maltophilia*; *S. pneumoniae*: *Streptococcus pneumoniae*.

Diagnosis of infections different from *H. capsulatum* or *P. jirovecii* was compiled from the medical records of the patients. Figure 1 depicted the concomitant infections within each study group. Several patients were infected with two or more different pathogens. The most important clinical and laboratory data regarding *H. capsulatum*, *P. jirovecii*, or co-infected patients were previously described in Carreto-Binaghi et al. [9].

### 3.1. Surfactant Protein and Cytokine Analyses of All Groups Studied

**Figure 2:**
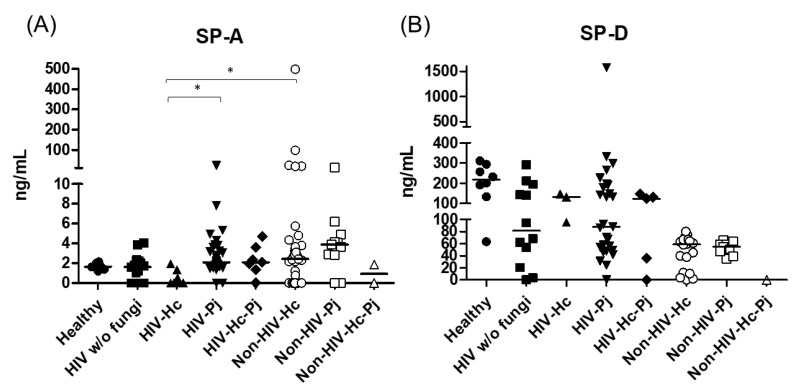
Surfactant proteins determination. Pulmonary SP-A and SP-D were measured in the BAL samples of hospitalized patients, using ELISA; results were obtained in ng/mL. Median values are shown for (**A**) SP-A and (**B**) SP-D. * *p* < 0.05.

Innate responses associated with SP-A and SP-D data measurements from each group of individuals are summarized in Table 2. SP-A median values were higher in most groups of infected patients (*H. capsulatum*, *P. jirovecii*, or co-infection) and statistical significance (*p* = 0.0007) was found among the eight groups of individuals studied (Figure 2). In general, the control groups (Healthy and HIV w/o fungi) showed similar SP-A values. In contrast to SP-A, SP-D values decreased in all patient groups when compared with the healthy controls, with no significance (*p* = 0.4397) among the eight groups studied (Figure 2).

**Figure 3:**
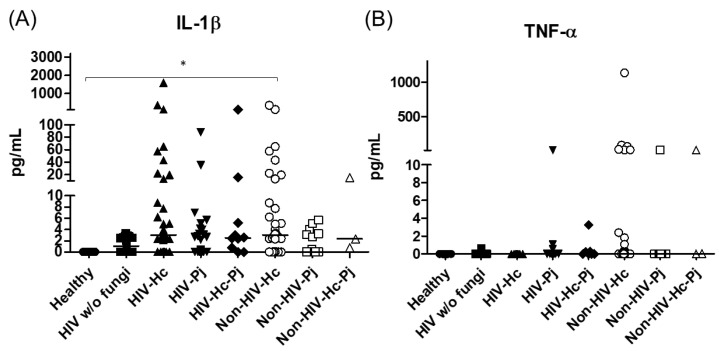
IL-1β and TNF-α proinflammatory cytokines determination. These cytokines were detected by a cytometric bead assay in the BAL samples of hospitalized patients; results were obtained in pg/mL. Median values are shown for (**A**) IL-1β and (**B**) TNF-α. * *p* < 0.05.

The analyzed pro- and anti-inflammatory cytokines are shown in Table 3. The proinflammatory mediators IL-1β and TNF-α increased significantly in all patient groups (*p* = 0.0002 and *p* = 0.0045, respectively) (Figure 3); however, in the individual analyses of TNF-α, there were no significant differences among the studied groups.

**Figure 4:**
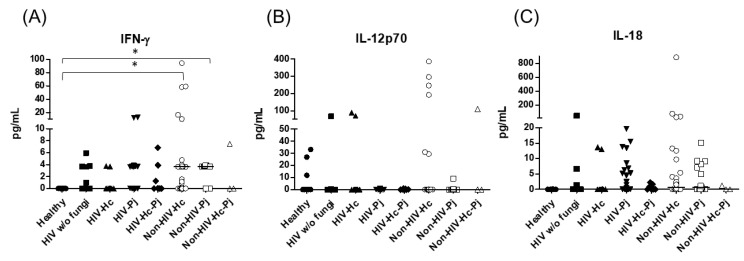
Determination of cytokines of the IFN-γ/IL-12 axis. IFN-γ, IL-12p70, and IL-18 were detected by a cytometric bead assay in the BAL samples of hospitalized patients; results were obtained in pg/mL. Median values are shown for (**A**) IFN-γ, (**B**) IL-12p70, and (**C**) IL-18. * *p* < 0.05.

Considering the role of the proinflammatory cytokines of the IFN-γ/IL-12 axis, we evaluated the involvement of IFN-γ and IL-12p70, as well as IL-18, and statistical analyses revealed significant differences for IFN-γ and IL-18 (*p* = 0.0003 and *p* = 0.0420, respectively) in the median values among all groups (Figure 4), even though individual comparisons for IL-18 did not show any differences. It is noteworthy that the non-HIV patient groups had the highest IFN-γ medians.

**Figure 5:**
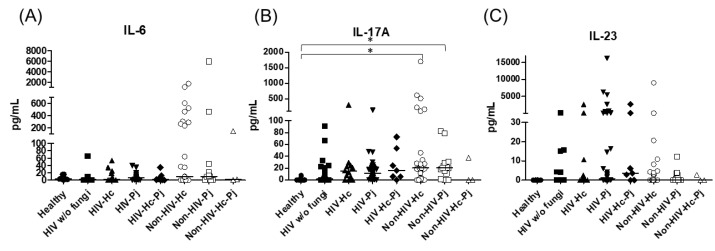
Determination of cytokines of the IL-17/IL-23 axis. IL-6, IL-17A, and IL-23 were detected by a cytometric bead assay in the BAL samples of hospitalized patients; results were obtained in pg/mL. Median values are shown for (**A**) Il-6, (**B**) IL-17A, and (**C**) IL-23. * *p* < 0.05.

**Figure 6:**
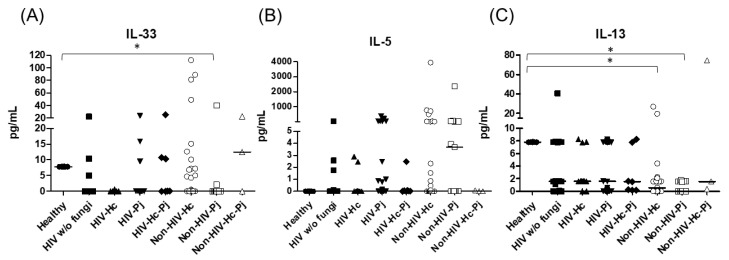
Determination of cytokines associated with granuloma formation. IL-33, IL-5, and IL-13 were detected by a cytometric bead assay in the BAL samples of hospitalized patients; results were obtained in pg/mL. Median values are shown for (**A**) IL-33, (**B**) IL-5, and (**C**) IL-13. * *p* < 0.05.

Regarding the proinflammatory cytokines of the IL-17/IL-23 axis, the involvement of IL-6, IL-17A, and IL-23 was analyzed, with a significant difference in IL-17A median values among all groups (*p* = 0.0118) (Figure 5). For IL-6 and IL-17A, it is remarkable that the non-HIV patient groups had higher values than the other groups (Figure 5). Among the cytokines related to granuloma formation, analysis of IL-33, IL-5, and IL-13 in the BAL samples showed significant differences in IL-33 and IL-13 among all groups (*p* = 0.0201 and *p* = 0.0018, respectively). Concerning IL-5, most non-HIV patient groups had high medians; however, no differences were found in the individual analyses of each group. In general, several patient groups showed decreased IL-13 median values compared with the control groups (Figure 6).

**Figure 7:**
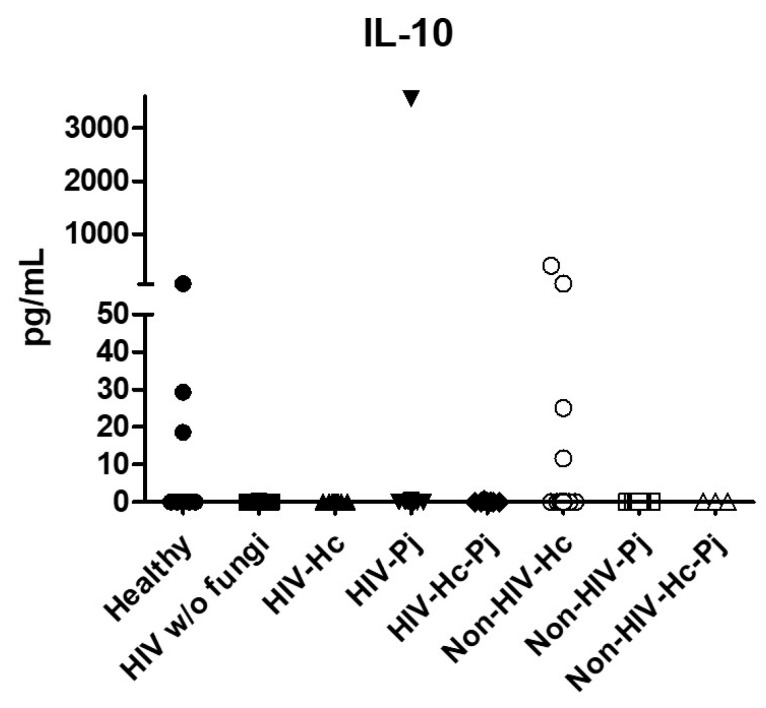
Determination of IL-10. This anti-inflammatory cytokine was detected by a cytometric bead assay in the BAL samples of hospitalized patients; results were obtained in pg/mL. Median values are shown.

Regarding the anti-inflammatory cytokine IL-10, results showed low values in most groups, although no statistical differences were found (Figure 7).

**Figure 8:**
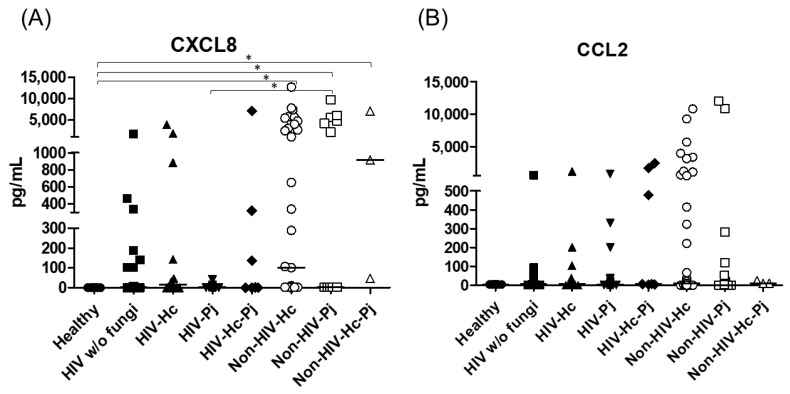
Determination of CXCL8 and CCL2 chemokines. These cytokines were detected by a cytometric bead assay in the BAL samples of hospitalized patients; results were obtained in pg/mL. Median values are shown for (**A**) CXCL8, and (**B**) CCL2. * *p* < 0.05.

The proinflammatory chemokines CXCL8 and CCL2 were also analyzed; a significant difference (*p* < 0.0001) in CXCL8 median values among all the studied groups was detected (Figure 8) and, interestingly, CXCL8 medians were higher in the non-HIV than in the HIV groups when compared with the Healthy control group (*p* < 0.05). No differences in the overall CCL2 values were found. Throughout the assays, the control groups always revealed undetectable or low pro- and anti-inflammatory cytokines median values.

### 3.2. Surfactant Protein and Cytokine Analyses Associated with H. capsulatum Infection

*H. capsulatum* infection induced a higher SP-A production in the Non-HIV-Hc group than in the HIV-Hc group (*p* < 0.05) (Figure 2). SP-D median values were consistently not significant in the individual comparisons between groups. A significantly higher median value (*p* < 0.05) for IL-1β was found when the Non-HIV-Hc group was compared with the healthy controls. In other patient groups with *H. capsulatum* infection, IL-1β values were also high (Figure 3), although no significance was reached. Likewise, the highest TNF-α median value in the Non-HIV-Hc group is also shown (see Figure 3), although no differences were found. A significant increase (*p* < 0.05) of IFN-γ was found in the Non-HIV-Hc group compared with that found in the healthy controls. Overall, patient groups infected with *H. capsulatum* (HIV-Hc and Non-HIV-Hc groups) showed higher IL-12p70 median values than the control groups, although no significance was reached (Figure 4). The Non-HIV-Hc group showed the highest IL-18 median value (Figure 4); however, individual comparisons between groups were not significant. IL-6 median values were higher in the Non-HIV-Hc group than in the control groups, although no significant differences were found (Figure 5). The individual analyses for IL-17A revealed significant differences (*p* < 0.05) between Non-HIV-Hc and healthy controls (Figure 5). Even though IL-23 displayed high median values in HIV-Hc and Non-HIV-Hc groups, no differences were found in the individual analysis for these groups (Figure 5). The highest IL-33 and IL-5 median values were shown in the Non-HIV-Hc group, while no significant differences were found (Figure 6). The Non-HIV-Hc group presented a significantly lower IL-13 median value than the healthy controls (*p* < 0.05) (Figure 6). Some patients from the Non-HIV-Hc group presented high values of the anti-inflammatory cytokine IL-10, although no statistical differences were found (Figure 7). The Non-HIV-Hc and HIV-Hc groups had higher CXCL8 median values than the healthy controls, but these values were only significant for the Non-HIV-Hc group (*p* < 0.05). CCL2 increased in the Non-HIV-Hc and HIV-Hc groups, although no significant differences were found in the individual analyses for these groups (Figure 8).

### 3.3. Surfactant Protein and Cytokine Analyses Associated with P. jirovecii Infection

In HIV patients, *P. jirovecii* infection (HIV-Pj group) induced a significantly higher SP-A production when compared with *H. capsulatum* infection (HIV-Hc group) (*p* < 0.05, Figure 2). However, the Non-HIV-Pj group developed a lower SP-A median value than the Non-HIV-Hc group, although no significant differences were found. The Non-HIV-Pj group induced a greater SP-A production compared with the HIV-Pj group (Figure 2); however, no significance was reached. As it occurred for *H. capsulatum* infection, SP-D median values were also consistently not significant in the individual comparisons between groups. IL-1β and TNF-α median values were slightly higher in the HIV-Pj and the Non-HIV-Pj groups than in the control groups; however, significance was not reached (Figure 3). In the non-HIV groups, *P. jirovecii* infection developed lower IL-1β and TNF-α medians than *H. capsulatum* infection, although significance was not reached either. A significant increase (*p* < 0.05) of IFN-γ was found in the Non-HIV-Pj group compared with the healthy controls (Figure 4). *P. jirovecii*-infected individuals (HIV-Pj and Non-HIV-Pj groups) had lower IL-12p70 median values than the control groups, as shown in Figure 4. Individual comparisons between groups did not show any differences for IL-18 median values. *P. jirovecii* infection (Non-HIV-Pj) developed lower IFN-γ, IL-12p70, and IL-18 median values than *H. capsulatum* infection (Non-HIV-Hc); besides, the HIV-Pj group also developed a lower median value of IL-12p70 than the HIV-Hc group (Figure 4), although no significant difference was found. The highest IL-6 median value was shown in the Non-HIV-Pj group; however, no significance was reached (Figure 5). IL-17A values were significantly higher in the Non-HIV-Pj than in the healthy controls (*p* < 0.05) (Figure 5). IL-23 displayed the highest median in the HIV-Pj group; however, no differences were found in this individual analysis (Figure 5). IL-17A and IL-23 also showed lower median values in the *P. jirovecii*-infected groups when compared with the *H. capsulatum*-infected groups, particularly in the non-HIV groups, although no significance was reached (Figure 5). A significantly higher difference (*p* < 0.05) was found between IL-33 median values from the Non-HIV-Pj and the healthy control group (Figure 6). Concerning IL-5, the HIV-Pj and the Non-HIV-Pj groups had higher median values when compared with the healthy controls; however, no differences were found. IL-13 values were significantly lower (*p* < 0.05) in the Non-HIV-Pj group than in the healthy controls (Figure 6). In general, in the non-HIV groups, *P. jirovecii* infection developed lower IL-33 and IL-13 medians than *H. capsulatum* infection; no significance was found(Figure 6). The HIV-Pj group showed the highest IL-10 value; however, no statistical differences were found when compared with the control groups (Figure 7). Significantly higher CXCL8 median values (*p* < 0.05) were found in the Non-HIV-Pj group when compared with the healthy controls and the HIV-Pj group (Figure 8). CCL2 also increased in the Non-HIV-Pj group, although no significant differences were found (Figure 8). In the HIV groups, *P. jirovecii* infection developed lower CXCL8 and CCL2 medians than *H. capsulatum* infection, although no significance was reached (Figure 8).

### 3.4. Surfactant Protein and Cytokine Analyses Associated with the Co-Infection of Both Pathogens

The co-infection group with a non-HIV condition (Non-HIV-Hc-Pj) showed a lower SP-A median value than the HIV-Hc-Pj group (Figure 2); however, no significance was reached. As inferred from the *H. capsulatum* and *P. jirovecii* infections, SP-D was always absent in co-infected groups. The Non-HIV-Hc-Pj and HIV-Hc-Pj groups developed higher IL-1β and TNF-α median values than the control groups, although no significant differences were found (Figure 3). However, in the non-HIV co-infected group these cytokines developed lower medians than those found for the Non-HIV-Hc group. Regarding IFN-γ, IL-12p70, and IL-18 analyses in co-infected groups, the Non-HIV-Hc-Pj group showed a higher IFN-γ median than the control groups, the highest IL-12p70 median value, and undetectable IL-18 (Figure 4); statistical analyses did not show differences in the individual comparisons between groups. Nevertheless, in this non-HIV co-infected group, IFN-γ and IL-18 developed lower median values than those found for the Non-HIV-Hc group. IL-6 median value in the Non-HIV-Hc-Pj group was higher than in the control groups; however, this co-infected group showed lower medians than the individual infections with both fungi; no significant differences were found for this cytokine (Figure 5). Regarding IL-17A, both co-infected groups (HIV and non-HIV) showed lower median values when compared with their respective individual *H. capsulatum* and *P. jirovecii* infected groups, highlighting the wider difference shown between the medians of Non-HIV-Hc and non-HIV co-infected group, although no significant differences were detected (Figure 5). IL-23 showed higher median values in the HIV-Hc-Pj group than in the control groups; however, no significance was reached (Figure 5). The Non-HIV-Hc-Pj group together with the healthy controls showed the lowest IL-23 median values, which contrast with the highest median associated with the Non-HIV-Hc group (Figure 5). IL-33 median values were higher in both co-infected groups (HIV and non-HIV) than in the *H. capsulatum* and *P. jirovecii* individual infection groups (Figure 6). These co-infected groups showed the lowest IL-5 medians, contrasting with higher median values of the Non-HIV-Hc and Non-HIV-Pj groups (Figure 6). Even though the Non-HIV-Hc-Pj group showed the highest IL-13 median value, no differences were found when compared with all groups (Figure 6). IL-10 values were undetectable in the Non-HIV-Hc-Pj and HIV-Hc-Pj groups (Figure 7). A significantly higher CXCL8 median value (*p* < 0.05) was found in the Non-HIV-Hc-Pj group when compared with the healthy controls (Figure 8). The HIV-Hc-Pj group had higher CXCL8 and CCL2 median values than the healthy controls, although significance was not reached (Figure 8). In contrast to CXCL8, the non-HIV co-infected group showed lower CCL2 median values than the individual infections with each fungus; no significant differences were found for this cytokine (Figure 8).

An interesting finding associated with the present results is the contrasting values of the inflammatory mediators (SP-A, IL-1β, TNF-α, IFN-γ, IL-18, IL-17A, and IL-23) of the non-HIV co-infected group, because co-infection induced lower median values of these cytokines, which were dissimilar to the values reached by individual Non-HIV-Hc or Non-HIV-Pj groups, highlighting that cytokines median values in *H. capsulatum* infection were always higher than in *P. jirovecii* infection. The response of other cytokines such as IL-6, IL-5, and CCL2, in the non-HIV groups showed lower median values in the *H. capsulatum*-infected group, in contrast to the higher values in the *P. jirovecii*-infected group; however, these cytokines remain low in the co-infected group.

### 3.5. Surfactant Protein and Cytokine Patterns Using a Heatmap Analysis

**Figure 9:**
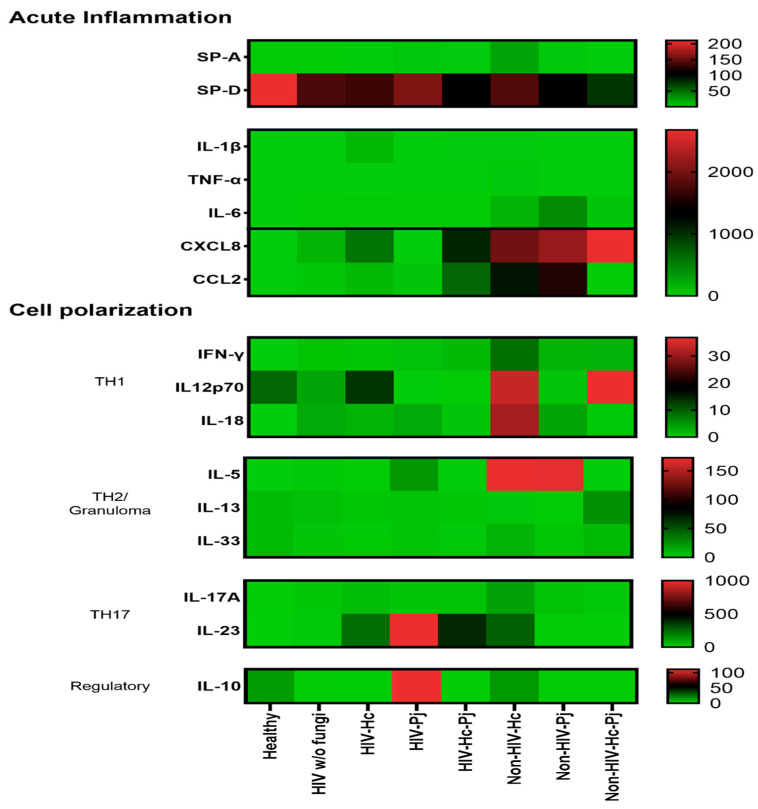
Heatmap of inflammatory mediators in *H. capsulatum* and *P. jirovecii* infections and their co-infection. Molecules were classified according to their primary immune function. Surfactant proteins and cytokines were clustered for the healthy control group, for each individual infection (*H. capsulatum* and *P. jirovecii*) and for co-infection. The heatmap shows the median concentration of each molecule per analyzed group; the scale is depicted at the right side of each module. Surfactant proteins were expressed in ng/mL and cytokines in pg/mL.

To validate collectin and cytokine results, a heatmap analysis was constructed considering the studied mediators associated with acute inflammation and cell polarization, using data of all studied groups regardless of the HIV condition (Figure 9). This analysis supports the data described in Figure 2, Figure 3, Figure 4, Figure 5, Figure 6, Figure 7 and Figure 8, which differs from one fungal pathogen to the other.

The patterns of SP-A, IL-1β, TNF-α, IL-6, and especially the chemokines (CXCL8 and CCL2) in the *H. capsulatum* patient group revealed the highest concentrations of these acute inflammatory mediators in contrast with their lower concentrations showed in *P. jirovecii* and co-infected groups (Figure 9). Among acute inflammatory mediators, SP-D developed a distinct behavior in all infected groups studied because its concentrations were always lower than those found in the healthy controls. The pattern of IL-12p70, IL-18, IL-5, and IL-17A in the *H. capsulatum* infection also revealed high concentrations of these cytokines involved in cell polarization, whereas their patterns in the *P. jirovecii* and co-infected groups were associated with low concentration values. The highest concentration of IL-23 was associated to *P. jirovecii* infection, although *H. capsulatum* and co-infected groups shared elevated concentrations of this cytokine (Figure 9). IL-10 revealed the highest concentration in *P. jirovecii* infection, which was not detected in the co-infected patients. Due to the minimal values of IFN-γ, IL-13, and IL-33, they were imperceptible in the heatmap analysis, as shown in Figure 9.

## 4. Discussion

Inflammatory mediators of the immune response to histoplasmosis and pneumocystosis have been described, considering the different settings of these mycoses and the distinct mammalian hosts [55,56]. For this study, with the aim to characterize the role of inflammatory mediators in histoplasmosis and pneumocystosis, we selected cytokines and collectins more frequently associated with these fungal infections. In addition, we assessed the presence of bacteria and viruses in the studied BAL fluid samples, and different mixed infections were registered together with the studied fungi.

Based on their critical roles in lung immunity, we investigated the levels of SP-A and SP-D in BAL samples from patients with *H. capsulatum* and *P. jirovecii* pneumonia and, for the first time, in patients co-infected with both fungi we searched for associations between the production of these collectins and these fungal infections.

The present results highlight a novel finding relating to a high SP-A production in non-HIV patients, mainly in those infected with *H. capsulatum* followed by those infected with *P. jirovecii*; the latter is consistent with previous reports on *Pneumocystis* sp. infection in rat [27] and mouse models [57], as well as humans [58]. Low SP-A levels in all HIV groups suggest an HIV-associated dysfunction in SP-A production or the presence of a high fungal burden, frequently observed in HIV/AIDS patients, where the SP-A molecules bind to the fungal surfaces, rendering SP-A undetectable. Interestingly, during co-infection SP-A levels decreased considerably, suggesting an in vivo antagonism of both fungi against SP-A induction or that SP-A molecules bound to the surfaces of each fungus, masking SP-A detection in BAL fluid. Here, the decreased SP-D median values in BAL samples, either in HIV or non-HIV patients, diverged from previous reports on lung fluid from HIV/AIDS patients without a pulmonary disease [59] and from scid/scid mouse pulmonary *P. carinii* (now *P. murina*) infection [57]. The lower levels of SP-D in the infected groups, in contrast to those found in the healthy controls, could be explained by the presence of basal SP-D levels in the host’s lungs [60,61], which might decrease during infections related to the proinflammatory status.

The involvement of pro- and anti-inflammatory cytokines, as well as cytokines associated with granuloma formation, was investigated in the collected BAL samples to identify distinct characteristics in human histoplasmosis or pneumocystosis pneumonia. To accomplish this goal, it was necessary to select the cytokines to be quantified in the Human Inflammation Panel assays (see Section 2).

Significant differences in the median values of IL-1β, TNF-α, IFN-γ, IL-18, IL-17A, IL-33, IL-13, and CXCL8 were reached in all the groups studied, suggesting that these cytokines play a role in the inflammatory processes associated with histoplasmosis and pneumocystosis.

In general, increased levels of IL-1β and TNF-α were found in non-HIV patients infected with *H. capsulatum*, which is interesting because IL-1β is important for the inflammasome formation [62]. This fungus is known to promote a Th1 response in murine models, favoring IL-1β, TNF-α, GM-CSF, and IFN-γ production in the host for *H. capsulatum* clearance [63,64]. The increased values of TNF-α in the Non-HIV-Hc group could be due to an acute lung inflammatory response to histoplasmosis [63,65,66].

The *H. capsulatum*-infected patient groups showed the highest median values for cytokines related to the IFN-γ/IL-12 axis, especially in the Non-HIV-Hc group. These results match the local cytokine production during a Th1 inflammatory response against *H. capsulatum* in non-HIV patients. Instead, lung inflammation in human pneumocystosis is part of a complicated chronic disease, mainly associated with HIV/AIDS patients [67,68]. In this study, among the cytokines of the IFN-γ/IL-12 axis, only IFN-γ increased in HIV and non-HIV *P. jirovecii*-infected groups. Although it is well known that Th1 response is absent in HIV patients, IFN-γ could also be produced by natural killer cells [69]. In the patient groups co-infected with both fungi, cytokine levels of the IFN-γ/IL-12 axis were low, suggesting an antagonistic interaction between *H. capsulatum* and *P. jirovecii* for their production.

Regarding cytokines of the IL-17/IL-23 axis, only IL-6 and IL-17 increased in the non-HIV patients infected with *H. capsulatum* and *P. jirovecii*. These findings correspond to earlier reports on IL-6 production in mice lung homogenates in response to *H. capsulatum* infection [70] and in A549-human cell line (type II pneumocytes) exposed to *Pneumocystis*’ proteins [71]; furthermore, the significant increase in IL-17A in these groups of patients suggest the induction of an inflammatory response. The highest IL-17A levels present in patient groups with histoplasmosis, mainly in the Non-HIV-Hc group, are in accordance with previous reports on the participation of Th17 response in experimental histoplasmosis infection [63,70]. A remarkable finding in our study was the significant production of IL-17A as part of the pulmonary immune response to *P. jirovecii* infection.

Cytokines involved in granuloma development, primarily IL-33, IL-5, and IL-13 were evaluated considering previous data on fungal infections [72,73]. In particular, IL-33 plays a critical role in granuloma formation during the late adaptive immune response to *H. capsulatum* in a murine model [74] and, based on our results, the Non-HIV-Hc group showed high IL-33 levels, supporting favorable conditions for granuloma development. In murine histoplasmosis infection, increased levels of IL-5 have been associated with *Histoplasma*-induced granulomas in the liver and the lung [75]. Here, we also described increased levels of IL-5 in BAL samples from the non-HIV histoplasmosis group, although significance was not reached. Despite the fact that granulomas are not related to *P. jirovecii* infection, a few case reports described atypical granulomatous reactions to pneumocystosis in non-HIV patients with distinct immunosuppression [76,77,78,79,80]. Increased serum IL-5 levels in human [81] and murine pneumocystosis [82] have been reported; here we found non-significant increased IL-5 levels in BAL samples from the *P. jirovecii*-infected groups (HIV and non-HIV). In the present study, significant IL-13 levels were detected in non-HIV patients with histoplasmosis and pneumocystosis, which could be related to IL-13 role in granuloma development, in contrast to the low levels reported in lung granulomas of *H. capsulatum*-infected mice [75].

The anti-inflammatory and regulatory IL-10 cytokine was detectable only in a few patients from the Non-HIV-Hc group, and damage involving this cytokine has been documented in *H. capsulatum* [63] and *P. jirovecii* [39] lung infections. A recent report by Rodriguez-Ramirez et al. [83] demonstrated that the presence of IL-10 inside coccidioidal granulomas might be associated with a fatal coccidioidomycosis outcome. According to our results, its absence in many BAL samples could be related to a favorable clinical-immunological status of the studied patients or because IL-10 production was inhibited by the presence of a macrophage hypoxia-inducible factor (HIF-1α) throughout *H. capsulatum* infection, as demonstrated by Fecher et al. [84].

The CXCL8 and CCL2 cytokines, also known as chemokines, were abundant in all the pneumonia groups. However, only CXCL8 levels were significantly high in the non-HIV histoplasmosis and pneumocystosis patients (Non-HIV-Hc, Non-HIV-Pj, and Non-HIV-Hc-Pj groups), possibly due to their association with local inflammatory reaction against these fungal infections. Our study shows, for the first time, CXCL8 in human histoplasmosis. Instead, when exposed to *Pneumocystis* surface proteins, the A549 human-pneumocyte cell line produces CXCL8 and CCL2 [85]. According to our results, most HIV-patients had low CXCL8 and CCL2 levels, a finding probably related to their HIV/AIDS status. In histoplasmosis murine models, CCL2 was associated with IL-4 production through a coordinated action of its CCR2-receptor [86], whereas in pneumocystosis murine models CCL2 and other cytokines (CCL3, CCL5, and cytokine-induced neutrophil chemoattractant) promoted a hyperinflammatory state, causing considerable lung damage after infection [45].

Finally, we inquired about the local cytokine production pattern in each evaluated group by displaying their median concentrations in a heatmap (see Figure 9). All groups of infected patients showed a high acute inflammatory response, whereas healthy controls produced only high amounts of SP-D. Particularly, chemokine-mediated *H. capsulatum*-infected patients showed a Th1/Th17 mixed pattern and *P. jirovecii*-infected patients displayed a clear Th17 polarization. In both infections, IL-5 could be contributing with the neutrophil function classically associated with the Th17 response [87], rather than promoting a Th2 environment.

Interestingly, the analyses of inflammatory mediator patterns (SP-A, IL-1β, IFN-γ, IL-18, and IL-17A) represented either in Figure 2, Figure 3, Figure 4, Figure 5, Figure 6, Figure 7 and Figure 8 or in Figure 9 (heatmap), as well as in Table 2 and Table 3, showed that *H. capsulatum* and *P. jirovecii* co-infection resembled *H. capsulatum* infection, although to a lesser extent, suggesting an immunomodulatory ability of *P. jirovecii* against *H. capsulatum* host response, irrespective of the HIV status. Infections caused by other pathogens could not modify these findings, as their distribution was similar in all groups of patients (Figure 1).

## 5. Conclusions

Our study identified a characteristic pulmonary collectin and cytokine pattern in *H. capsulatum*, *P. jirovecii*, or co-infection pneumonia not reported before in this type of patients, irrespective of their HIV condition. Moreover, our results suggest an interesting antagonistic effect of *P. jirovecii* against the *H. capsulatum*-driven immune response when both fungi coexist in the host. However, being able to precisely determine the time of infection in humans remains a challenge; hence, this uncertainty might contribute to data interpretation bias. Although for *P. jirovecii* molecular diagnosis was used in view of its high sensitivity (97–99%) and specificity (90–94%), as mentioned by Bateman et al. [12], we acknowledge that this type of diagnostic tool does not separate colonization and disease to-date; however, when molecular results match with medical data of pulmonary injury, molecular diagnosis reinforces the clinical diagnosis. Since all patients in our study were hospitalized with an acute hypoxemic pneumonia diagnosed based on clinical grounds and supported by the molecular approach for the studied fungi, pulmonary colonization was considered less likely.

It is pertinent to mention that an unavoidable limitation of this study was the potential variability between each patient, considering the degree of immunocompromise, comorbidities, and the drugs they were being treated with before hospitalization, all of which could modulate their immune response. Further studies are required to elucidate the role of the immune response in the pathogenesis of pneumonia with *H. capsulatum* and *P. jirovecii* co-infection.

## Figures and Tables

**Table 1:** Studied groups of individuals. Abbreviations in parenthesis are the same in all figures.

Group	Number of Individuals
Healthy volunteers (Healthy, control)	8
HIV-positive without the studied fungi (HIV w/o fungi, control)	19
HIV-positive with histoplasmosis (HIV-Hc)	12
HIV-positive with pneumocystosis (HIV-Pj)	32
HIV-positive with co-infection (HIV-Hc-Pj)	7
HIV-negative with histoplasmosis (Non-HIV-Hc)	35
HIV-negative with pneumocystosis (Non-HIV-Pj)	15
HIV-negative with co-infection (Non-HIV-Hc-Pj)	3
TOTAL	131

**Table 2:** Detection of SP-A and SP-D. Median values for all group determinations are shown in ng/mL, followed by their corresponding 25–75 interquartile range in parenthesis. Abbreviations of each group of individuals are the same in all figures. The presence of SP-A and SP-D was determined by separate BioAssay ELISA Kits in BAL samples of hospitalized patients (details in the Section 2).

Group of Individuals	SP-A	SP-D
Healthy, control	1.654 (1.452–1.966)	217.0 (147.4–284.8)
HIV w/o fungi, control	1.593 (0–2.243)	140.3 (53.91–228.2)
HIV-Hc	0 (0–0.4048)	139.6 (48.28–192.9)
HIV-Pj	2.077 (1.538–3.505)	87.01 (46.15–196.2)
HIV-Hc-Pj	2.105 (1.351–3.602)	123.0 (35.95–147.5)
Non-HIV-Hc	2.425 (0–3.785)	79.96 (58.36–253.9)
Non-HIV-Pj	2.889 (0–4.175)	58.91 (47.04–219.7)
Non-HIV-Hc-Pj	0 (0–1.859)	0 (0–227.1)

**Table 3:** Detection of cytokines. Median values for all group determinations are shown in pg/mL, followed by their corresponding 25–75 interquartile range in parenthesis. Abbreviations of each group of individuals are the same in all figures. The presence of cytokines was determined using the LEGENDPLEX Human Inflammation Panel (BioLegend) in BAL samples of hospitalized patients (details in the Section 2).

Cytokines	Healthy (Control)	HIV w/o Fungi (Control)	HIV-Hc	HIV-Pj	HIV-Hc-Pj	Non-HIV-Hc	Non-HV-Pj	Non-HIV-Hc-Pj
IL-1β	Undetectable	1.03 (0–2.58)	2.90 (0–3.58)	0 (0–0.15)	2.56 (0–5.22)	3.01 (0–8.74)	0 (0–3.27)	2.34 (0.74–15.53)
TNF-α	Undetectable	0 (0–0)	Undetectable	0 (0–0)	0 (0–0.25)	0 (0–1.09)	0 (0–0)	0.04 (0–14.18)
IFN-γ	Undetectable	0 (0–0.99)	0 (0–0)	0 (0–0)	0 (0–3.87)	3.62 (0–3.82)	3.66 (0–3.74)	0 (0–7.49)
IL-12p70	0 (0–23.15)	0 (0–0.01)	0 (0–0.20)	0 (0–0.03)	0 (0–0.57)	0 (0–0.20)	0 (0–0.17)	0.06 (0–110.50)
IL-18	Undetectable	0 (0–0.33)	0 (0–0)	0 (0–5.22)	0 (0–1.62)	0.55 (0–4.10)	0.71 (0–8.12)	0 (0–1.21)
IL-6	3.38 (0–12.80)	2.02 (0–9.09)	1.47 (0–23.70)	6.80 (0–9.91)	1.41 (0–12.54)	9.13 (0.88–232.00)	10.09 (0–21.07)	1.77 (0–140.80)
IL-17	0.03 (0–0.64)	1.80 (0–22.69)	14.83 (1.53–24.25)	11.25 (3.58–21.49)	15.93 (5.71–53.56)	20.39 (3.59–28.15)	20.65 (10.91–28.87)	0 (0–37.99)
IL-23	Undetectable	0 (0–4.11)	0 (0–8.64)	0.77 (0–54.78)	3.61 (0–120.10)	0 (0–3.18)	0 (0–2.84)	0.65 (0–2.79)
IL-33	7.80 (7.78–7.82)	0.11 (0–7.81)	0.04 (0–5.97)	0.39(0–7.82)	0.16 (0–7.77)	0 (0–5.04)	0 (0–0)	0.36 (0–22.73)
IL-5	Undetectable	0 (0–0.09)	0 (0–0)	0 (0–0.95)	0.03 (0–0.07)	0.00 (0–2.31)	3.68 (0–29.91)	0 (0–0.08)
IL-13	7.80 (7.78–7.82)	1.58 (1.12–7.83)	1.59 (1.56–6.24)	1.56 (0–7.82)	1.53 (0.16–7.77)	0.57 (0–1.58)	0 (0–1.57)	1.53 (0.36–74.67)
IL-10	0 (0–26.67)	0 (0–0)	Undetectable	0 (0–0)	0 (0–0.12)	0 (0–0)	0 (0–0)	0 (0–0.01)
CXCL8	Undetectable	2.29 (0–140.60)	16.33 (0–699.90)	1.94 (0–2.29)	1.35 (0–319.40)	101.20 (2.28–4018.00)	2.40 (2.17–4749.00)	916.60 (46.52–7072.00)
CCL2	4.47 (3.99–5.04)	5.35 (0.79–13.73)	6.47 (0.46–88.25)	4.16 (0–9.94)	6.69 (3.69–1700.00)	9.03 (0–650.90)	0 (0–118.10)	11.35 (10.40–23.73)

## Data Availability

The data presented in this study are available within the article.
